# A gene expression signature predicts recurrence-free survival in meningioma

**DOI:** 10.18632/oncotarget.24498

**Published:** 2018-02-15

**Authors:** Adriana Olar, Lindsey D. Goodman, Khalida M. Wani, Nicholas S. Boehling, Devi S. Sharma, Reema R. Mody, Joy Gumin, Elizabeth B. Claus, Frederick F. Lang, Timothy F. Cloughesy, Albert Lai, Kenneth D. Aldape, Franco DeMonte, Erik P. Sulman

**Affiliations:** ^1^ Medical University of South Carolina & Hollings Cancer Center, Departments of Pathology and Laboratory Medicine & Neurosurgery, Charleston, SC, USA; ^2^ Neurosciences Graduate Group, Perlman School of Medicine, University of Pennsylvania, Department of Biology, Philadelphia, PA, USA; ^3^ The University of Texas MD Anderson Cancer Center, Department of Translational Molecular Pathology, Houston, TX, USA; ^4^ St. Charles Cancer Center, Department of Radiation Oncology, Bend, OR, USA; ^5^ The University of California at Los Angeles, Department of Neurology, David Geffen School of Medicine, Los Angeles, CA, USA; ^6^ The University of Texas MD Anderson Cancer Center, Department of Neurosurgery, Houston, TX, USA; ^7^ Brigham and Women’s Hospital, Harvard Medical School, Department of Neurosurgery, Boston, MA, USA; ^8^ School of Public Health, Yale University, Department of Biostatistics, New Haven, CT, USA; ^9^ MacFeeters-Hamilton Brain Tumour Centre, Princess Margaret Cancer Centre, Toronto, Ontario, Canada; ^10^ The University of Texas MD Anderson Cancer Center, Departments of Radiation Oncology and Genomic Medicine, Houston, TX, USA

**Keywords:** meningioma, gene expression, affymetrix, recurrence risk, predictor algorithm

## Abstract

**BACKGROUND:**

Meningioma is the most common primary brain tumor and has a variable risk of local recurrence. While World Health Organization (WHO) grade generally correlates with recurrence, there is substantial within-grade variation of recurrence risk. Current risk stratification does not accurately predict which patients are likely to benefit from adjuvant radiation therapy (RT). We hypothesized that tumors at risk for recurrence have unique gene expression profiles (GEP) that could better select patients for adjuvant RT.

**METHODS:**

We developed a recurrence predictor by machine learning modeling using a training/validation approach.

**RESULTS:**

Three publicly available AffymetrixU133 gene expression datasets (GSE9438, GSE16581, GSE43290) combining 127 primary, non-treated meningiomas of all grades served as the training set. Unsupervised variable selection was used to identify an 18-gene GEP model (18-GEP) that separated recurrences. This model was validated on 62 primary, non-treated cases with similar grade and clinical variable distribution as the training set. When applied to the validation set, 18-GEP separated recurrences with a misclassification error rate of 0.25 (log-rank p=0.0003). 18-GEP was predictive for tumor recurrence [p=0.0008, HR=4.61, 95%CI=1.89-11.23)] and was predictive after adjustment for WHO grade, mitotic index, sex, tumor location, and Simpson grade [p=0.0311, HR=9.28, 95%CI=(1.22-70.29)]. The expression signature included genes encoding proteins involved in normal embryonic development, cell proliferation, tumor growth and invasion (FGF9, SEMA3C, EDNRA), angiogenesis (angiopoietin-2), cell cycle regulation (CDKN1A), membrane signaling (tetraspanin-7, caveolin-2), WNT-pathway inhibitors (DKK3), complement system (C1QA) and neurotransmitter regulation (SLC1A3, Secretogranin-II).

**CONCLUSIONS:**

18-GEP accurately stratifies patients with meningioma by recurrence risk having the potential to guide the use of adjuvant RT.

## INTRODUCTION

Meningioma is the most common primary brain tumor accounting for approximately 36% of all primary central nervous system tumors [1]. Based on histologic criteria, meningioma is currently classified into three World Health Organization (WHO) grades (I, II or atypical, and III or anaplastic) [2]. Although overall survival for patients with meningioma is usually prolonged (years) these tumors frequently cause severe patient morbidity and decreased quality of life [3, 4]. The current treatment options are surgical resection, followed by serial imaging to monitor for tumor recurrence and/or adjuvant radiation therapy (RT) based on tumor location, size, extent of surgical resection, and WHO grade [5]. WHO grade along with the extent of surgical resection (assessed by the operating surgeon and recorded as the Simpson grade [6]) are the strongest predictors of tumor recurrence [6–8]. While the WHO grade correlates with recurrence (WHO I, II, and III recur in up to 20%, 40%, and 50-90% respectively) there is substantial within-grade variation of recurrence risk [2, 9–12]. Tumor recurrence requires further treatment and may warrant adjuvant RT following surgical resection [5]. The current risk stratification system that is mainly based on WHO grade is imprecise [2, 13]. That means that the WHO grade is not able to accurately identify patients prone to meningioma recurrence. For example patients with WHO grade I tumors could experience early tumor recurrence and very aggressive disease course, while patients with WHO grade II tumors could not. This creates difficulty clinically in deciding whom to offer RT; therefore, patients who are likely to benefit from adjuvant RT are not accurately identified. Similarly, patients not at risk for tumor recurrence, if accurately identified could be spared the potential toxicity of RT.

In an effort to find a recurrence risk classifier that could improve over WHO and Simpson grades, we hypothesized that gene expression profiles (GEP) correlate with meningioma recurrence and could be used to better stratify patients both for determining follow-up interval and for RT. To test this hypothesis we used 3 publicly available Affymetrix gene expression datasets consisting of 127 patients with primary, non-treated meningiomas to identify an expression model that we further validated on 62 new patients with primary, non-treated tumors from M.D Anderson Cancer Center. We identified an 18-gene GEP classifier (18-GEP) that accurately stratified patients with meningioma by recurrence risk.

## RESULTS

The training dataset consisted of a cohort of 127 publicly available samples including 92 (73%) WHO I, 32 (25%) WHO II, and 2 (2%) WHO III meningiomas. The median follow-up was 5.53 years (range 0.05-25.42) and 18 patients experienced tumor recurrence. Detailed clinical characteristics of the training dataset are provided in Table 1. Baseline survival analysis by WHO grade is provided in Supplementary Figure 1. The validation dataset (consisting of 62 samples from M.D. Anderson Cancer Center) included 30 (48.39%) WHO I and 32 (51.61%) WHO II meningiomas. Most patients were females with a F:M ratio of 2.26 and the median age at initial diagnosis was 56.77 years (range 11.2-86.48). Median follow-up time for the validation dataset was 5.19 years (range 0.27-19.99). Most tumors were located in the skull base (skull base vs. non skull base ratio=2.44). Almost half of patients underwent Simpson 1 grade surgical resection (45.16%). One chordoid and 1 secretory meningioma were included. Most tumors had low mitotic (≤2, 58.68%) (58/62 interrogated) and MIB-1 (≤5, 56.45%) (all interrogated) indices. Twelve patients experienced tumor recurrence. Detailed clinical characteristics of the validation dataset are provided in Table 2.

**Table 1:** Clinical characteristics of the training dataset^*^

Publicly available dataset [Ref.]	WHO I	WHO II	WHO III	Total WHO	Median F/U (range) (years)
**Recurrences/n**					
**GSE9438** [37]	2/24^**^	2/6	-	4/31	9.02 (2.66-10.33)
GSE16581^***^[45]	2/35	3/13	1/1	6/49	5.48 (0.05-8.80)
GSE43290^****^[38]	3/33	4/13	1/1	8/47	4.67 (1.42-25.42)
**Recurrences/Total (%)**	7/92 (73)	9/32 (25)	2/2 (2)	18/127 (100)	5.53 (0.05-25.42)

Legend: F/U, follow-up; n, number of cases; Ref., reference; WHO, World Health Organization.

^*^ Only this information was uniformly available for all cases.

^**^ The WHO grade was not available for one case.

^***^ 19 samples were eliminated due to the following reasons: 3 samples lacked clinical data, 9 samples were recurrent tumors, 7 samples lacked time to progression data.

^****^ 4 normal meningeal cell samples were eliminated.

**Table 2:** Clinical characteristics of the validation dataset (n=62)

WHO	
Recurrences/n (%)	
I	5/30 (48.39)
II	7/32 (51.61)
III	N/A
**Median F/U** (range) (years)	5.19 (0.27-19.99)
**Median age** (range) (years)	56.77 (11.2-86.48)
**Simpson grade** n (%)	
**1**	28 (45.16)
**2**	16 (25.81)
**3**	4 (6.45)
**4**	11 (17.74)
**N/A**	3 (4.84)
**Mitotic index** n (%)	
**≤ 2**	37 (58.68)
**3-4**	13 (20.97)
**≥5**	8 (12.90)
**N/A**	4 (6.45)
**Median MIB-1 index** (range)	3.65 (0.2-20)
**Sex** n (%)	
**F**	43 (69.35)
**M**	19 (30.65)
**Location** n (%)	
**Skull-base**	44 (70.97)
**Non skull-base**	18 (29.03)

Legend: F, female; F/U, f ollow-up; M, male; n, number of cases; N/A, not available or not applicable; WHO, World Health Organization.

**Figure 1:**
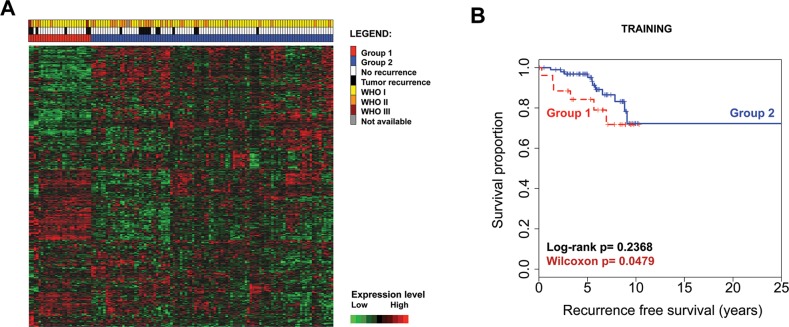
Unsupervised hierarchical clustering with the initial filtered 393 probe sets in the training dataset (n=127) separates 2 differentially expressed groups of tumors. Each row represents a probe set and each column represents a sample. Expression values are shown after batch normalization **(A)**. Kaplan–Meier survival analysis illustrates a trend for decreased tumor recurrence time for patients with meningioma from Group 1 and early survival curve separation **(B)**.

The training dataset expression data was first analyzed in order to identify an expression model that could better classify patients by meningioma recurrence. After the optimal model was selected, it was applied to the validation dataset. Following data import and batch normalization, the probe sets with a median absolute deviation (MAD) score ≥ 0.5 (n=491) underwent k-means clustering filtering and 393 probes were further kept in the analysis. Unsupervised hierarchical clustering of these 393 probe sets separated 2 groups that showed a trend towards separating risk categories (Wilcoxon p=0.0479) (Figure 1A and 1B); however only 5/18 recurrences were classified in the poor prognostic group (Group 1 in Figure 1). Similarly, when the same 393 probe sets were clustered hierarchically in the validation dataset (comprising 62 samples from our institution), similar expression groups emerged but with weak separation of the associated survival curves (Supplementary Figure 2).

**Figure 2:**
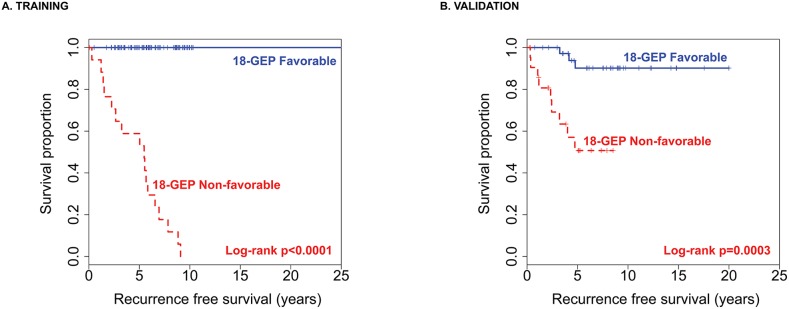
The 18-GEP model applied to the training dataset **(A)** and then to the validation dataset **(B)** significantly separates risk groups for meningioma recurrence.

In order to find a more optimal model and improve classification in the poor prognostic group, the 393 probe sets were introduced in a support vector machine classification method with radial-basis smoothing kernel (RBM) prediction algorithm, using the deviance residual as the continuous dependent variable. Unsupervised variable selection followed by RBM resulted in a final 18 GEP model with a 50-fold cross-validation root mean square error (RMSE) of 0.17. Partition analysis of the inverse transformed predictor probabilities returned a cutoff of 0.3608 that separated risk groups. Predictor probabilities > 0.3608 (18-GEP Non-favorable class) separated 17/18 recurrences and patients categorized here had significantly decreased median RFS (5.48 years, range: 0.05-9.08) compared to those with predictor probabilities ≤ 0.3608 (18-GEP Favorable class) (median RFS=25.42 years, range:0.52-25.42) (Log-rank p<0.0001) (Figure 2A).

The 18-GEP classifier was then applied to the validation dataset and the 18-GEP Non-favorable class separated 9/12 recurrences with a misclassification error rate of 0.25 (log-rank p=0.0003) (Figure 2B). The 18-GEP prognosticator was significantly predictive of tumor recurrence independently (p <<0.0001, HR=6.55, 95%CI:3.18-13.49) and when adjusted for WHO grade in the training dataset (p<<0.0001, HR=7.12, 95%CI:2.96-17.11) (Table 3). Similarly, the 18-GEP was significantly predictive of tumor recurrence (p=0.0008, HR=4.61, 95%CI:1.89-11.23) and when adjusted for WHO grade, mitotic index, Simpson grade, sex, and tumor location (p=0.0311, HR=9.28, 95%CI:1.22-70.29) (Table 4).

**Table 3:** Cox univariate and multivariate analyses for the training dataset (n=127)

Variable	Univariate			Multivariate^*^		
	HR	95% CI	P-value	HR	95% CI	P-value
**18-GEP**	6.55	3.18-13.49	≪0.0001	7.12	2.96-17.11	≪0.0001
WHO^*^						
II vs. I	4.15	1.49-11.54	0.0065	3.48	1.12-10.86	0.0314
III vs. I	358.25	29.40-4365.04	≪0.0001	85.06	6.55-1104.08	<0.0001

Legend: 18-GEP, 18 gene expression profile model; CI, confidence interval; HR, hazard ratio.

^*^126 cases with complete data were introduced in the model.

**Table 4:** Cox univariate and multivariate analyses for the validation dataset (n=62)

Variable	Univariate			Multivariate^*^		
	HR	95% CI	P-value	HR	95% CI	P-value
**18-GEP**	4.61	1.89-11.23	0.0008	9.28	1.22-70.29	0.0311
**WHO**						
II vs. I	1.39	0.44-4.37	0.577	0.53	0.09-3.11	0.4857
Mitotic index^**^						
3-4 vs. ≤ 2	1.24	0.23-6.78	0.8034	0.74	0.05-10.70	0.8228
≥5 vs. ≤ 2	5.29	1.32-21.23	0.0187	24.67	3.40-179.06	0.0015
Simpson grade^***^						
2 vs. 1	3.62	0.66-19.76	0.1378	1.45	0.17-12.52	0.7364
3 vs. 1	10.51	1.47-74.91	0.0189	2.96	0.08-107.56	0.5533
4 vs. 1	5.11	0.93-27.90	0.0599	4.54	0.60-34.08	0.1416
**Sex**						
M vs. F	2.08	0.67-6.45	0.205	0.46	0.06-3.69	0.4619
**Location**						
Skull-base vs. Non skull-base	1.84	0.58-5.82	0.298	1.14	0.17-7.76	0.8971

^*^ 55 cases with complete data across all variables were introduced in the model.

^**^58 cases with complete data were introduced in the univariate model.

^***^59 cases with complete data were introduced in the univariate model.

Legend: 18-GEP, 18 gene expression profile model; CI, confidence interval; F, female; HR, hazard ratio; M, male.

Three out of 4 meningioma patients who received remote radiation to the skull for other causes (WHO grades I, I, and II, of which a single WHO grade I tumor recurred after 3.23 years) classified as 18-GEP Favorable. The remaining meningioma (WHO grade II, non-recurrent), classified as 18-GEP Non-favorable.

The 18 expression signature genes identified are represented in Table 5.

**Table 5:** The 18 expression signature genes

#	Gene symbol	Location	Official name/description
*1*	*ANGPT2*	8p23.1	angiopoietin 2
*2*	*C1QA*	1p36.12	complement component 1, q subcomponent, A chain
3	*CAV2*	7q31.2	caveolin 2
4	*CDKN1A*	6p21.2	cyclin-dependent kinase inhibitor 1A (p21, Cip1)
5	*DKK3*	11p15.3	dickkopf WNT signaling pathway inhibitor 3
6	*EDNRA*	4q31.22	endothelin receptor type A
7	*EXT1*	8q24.11	exostosin glycosyltransferase 1
8	*FGF9*	13q12.11	fibroblast growth factor 9
9	*FMO2*	1q24.3	flavin containing monooxygenase 2
10	*LAMP5*	20p12.2	lysosomal-associated membrane protein family, member 5
11	*MLPH*	2q37.3	melanophilin
12	*PHLDA2*	11p15.4	pleckstrin homology-like domain, family A, member 2
13	*RHOBTB3*	5q15	Rho-related, broad-complex, tramtrack and bric à brac domain containing 3
14	*RUNDC3B*	7q21.12	RUN domain containing 3B
15	*SCG2*	2q36.1	secretogranin II
16	*SEMA3C*	7q21.11	sema domain, immunoglobulin domain, short basic domain, secreted or semaphoring 3C
17	*SLC1A3*	5p13.2	solute carrier family 1 (glial high affinity glutamate transporter), member 3
18	*TSPAN7*	Xp11.4	tetraspanin 7

**Figure 3:**
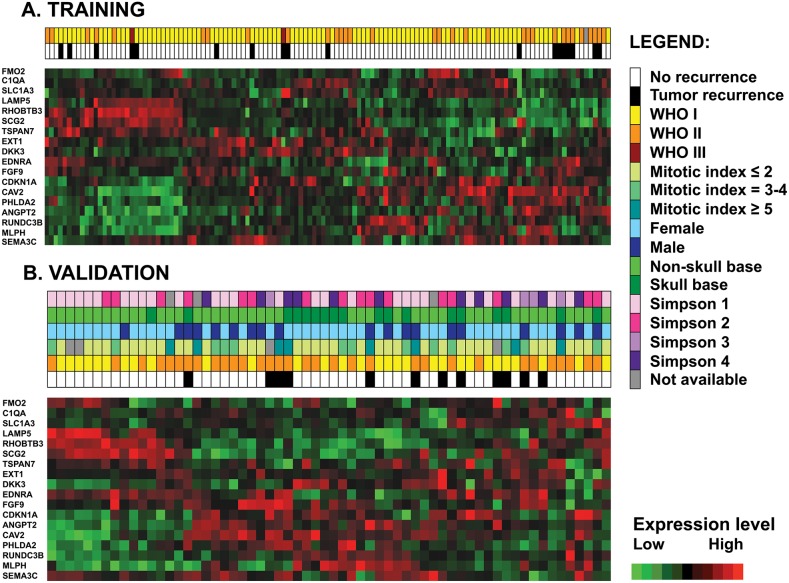
Unsupervised hierarchical clustering with the 18 model probe sets in the training dataset (n=127) **(A)** and in the validation dataset **(B)** shows similar patterns of gene expression. Each row represents a probe set and each column represents a sample. Expression values are shown after batch normalization.

Unsupervised hierarchical clustering using only these 18 probe sets in both training and validation datasets yielded similar expression profiles (Figure 3). Six of these genes were usually underexpressed (*RUNDC3B, ANGPT2, PHLDA2, CAV2, EDNRA*, and *SCG2)* in non-aggressive/non-recurrent tumors and overexpressed in more aggressive/recurrent meningiomas. Four genes were usually hyperexpressed (*FMO2, MLPH, RHOBTB3, LAMP5)* in non-recurrent tumors and underexpressed in more aggressive/recurrent tumors.

## DISCUSSION

We show that recurrent meningiomas have unique GEP that could be used to better stratify patients for postoperative management. Our 18-gene expression predictor is able to accurately identify patients who will experience tumor recurrence and therefore has the potential to guide therapy. Indeed our predictor correctly classified 9/12 (75%) meningioma recurrences in the validation set and remained statistically significant after adjustment for WHO and Simpson grades, the most powerful recognized predictors to date [6–8]. This indicates that molecular diagnostics, including gene expression, should be used in addition to histologic grading and extent of resection to estimate meningioma recurrence risk for individual patients. This would help inform decisions regarding follow-up intervals and the need for postoperative RT.

The 18 genes identified in this profile warrant further investigation. The expression signature included genes encoding proteins involved in normal embryonic development, cell proliferation, tumor growth and invasion (FGF9, SEMA3C, EDNRA) [14–16], angiogenesis (ANGPT2) [17], cell cycle regulation (CDKN1A) [18], membrane signaling (TSPAN7, CAV2) [19, 20], WNT-pathway inhibitors (DKK3) [21, 22], complement system (C1QA) [23] and neurotransmitter regulation (SLC1A3, SCG2) [24, 25].

By visual inspection of the 18-GEP heatmaps in both datasets (Figure 3) supported by quantitation of gene expression levels (Supplementary Table 1) a group of six genes (*RUNDC3B, ANGPT2, PHLDA2, CAV2, EDNRA*, and *SCG2)* seemed to be usually overexpressed and a group of four genes (*FMO2, MLPH*, *RHOBTB3, LAMP5*) seemed to be usually underexpressed in recurrent tumors. Of these, several have been previously investigated in meningioma and linked to meningioma tumorigenesis, either in small-size or in large-scale expression profiling studies. Most studies reported evidence supporting an angiogenetic mechanism for meningioma formation/progression. Ilhan et al. showed that patients with meningioma had high plasma concentrations of the proangiogenic factor ANGPT2 (even higher than patients with glioblastoma) but they did not report a correlation with survival [17]. Although, *CAV2* has not been investigated in meningioma, increased CAV1 protein immunoexpression, a family member of the caveolin family, has been reported in meningioma and associated with an increased MIB-1 index and poor prognosis [19] while *CAV1* has been reported as downregulated in WHO grade I meningioma [26]. Importantly, CAV1 was shown to correlate with increased angiogenesis in meningioma [27]. Similarly, SEM3C has not been previously studied in meningioma, elevated immunoexpression levels of its class member SEM3A, an antiangiogenic factor, has been associated with decreased meningioma recurrence [28]. It is likely that an angiogenetic program contributes at least in part to meningioma formation and progression and more in-depth investigational studies should focus into understanding these underlying mechanisms in order to design appropriate and accurate targeted therapeutic agents. This would be especially beneficial to patients prone to meningioma recurrence or those with aggressive grade tumors.

Some studies have shown that levels of CDKN1A (p21) immunoexpression correlated with MIB-1 immunoexpression in meningioma [18, 29] and increased with increasing tumor grade [18, 29, 30]. Others have shown that *CDKN1A* is downregulated in less aggressive meningiomas, especially fibroblastic meningioma [31]. On the other hand, others reported increased nuclear protein expression levels in benign meningiomas [32, 33]. Irrespective of their conclusions it seems that *CDKN1A* has an important role in meningioma growth and proliferation.

*DKK3* has not been described in meningioma, but was reportedly downregulated in malignant glioma and its expression had anti-tumor effects in glioblastoma *in vivo* and *in vitro* (increased expression blocks the WNT signaling pathway, decreased expression activates it) [21, 22]. Interestingly here we observed that *DKK3* was mainly overexpressed in aggressive meningiomas. While this needs confirmation by other studies it is possible that other, non-WNT signaling pathways are triggered in meningioma.

*MLPH*, encoding a G-protein with roles in melanosome transport, has been shown to be differentially expressed in less aggressive (*benign*) histologic variants of meningioma. More exactly it is upregulated in meningothelial meningioma and downregulated in fibroblastic variants [34]. Our data show that *MLPH* underexpression was usually associated with more aggressive meningiomas. Further studies should further confirm these findings and investigate the potential role the encoded protein might have in meningioma progression.

Interestingly, increased mRNA and EDNRA protein expression has been systematically described and associated with tumorigenesis in meningiomas [15, 35]. A statistically significant difference between *EDNRA* expression in benign versus WHO II–III meningiomas with higher gene expression levels in more aggressive tumors has also been reported [36]. In contrast *EDNRA* upregulation was reported by Claus et al (in a dataset including in the training samples of the current study) and associated with progesterone receptor positive meningiomas, which were reported to be usually low grade, non-aggressive tumors, with lower rates of recurrence [37]. Similarly, *TSPAN7* downregulation has been reported in more aggressive types of meningioma [34]. Tabernero et al reported *RHOBTB3* (encoding a GTP-ase) overexpression in meningiomas with monosomy 22/−22q alone (in a dataset including the training samples of the current study) [38].

While *PHLDA2* has not been previously described in meningioma, its class member, PHLDA1 was reportedly overexpressed in aggressive meningioma [39]. From the other 18-GEP genes, FGF9 has been consistently shown to be secreted and immunoexpressed in meningioma tissues [16]. FGF9 underexpression was associated with aggressive meningiomas [39] and gene overexpression with WHO grades I and II meningiomas [26, 40], especially the fibroblastic morphological subtype [31]. Interestingly, C1QA complement component has been recently reported as overexpressed in WHO I and II meningiomas [40]. While C1QA complement component has not been intensely studied in meningioma, different levels of complement regulatory membrane proteins, particularly CD55 and CD59, have been reported in meningioma by several groups. Shinoura et al. reported low levels of CD55/CD59 by Northern blot analysis in meningioma tissues [41] while Domingues et al. reported high levels of these regulatory proteins in meningioma cell membranes by flow cytometry and Affymterix GEP [42, 43] that might be associated with tumor response to complement cytotoxicity [43]. Based on the literature and supported by our findings, complement activation with tissue deposition of complement system products in meningioma tissues may occur, as has been reported in glioblastoma [23]. *RUNDC3B, SCG2, SLC1A3, EXT1* (as well as *RHOBTB3, TSPAN7, CAV2, MLPH)* were part of the *NF2/SMARCB1* expression subclass of a recent study on genomic profiling of meningioma. On a cohort of 79 primary, non-radiated WHO grade I meningiomas, Clark et al. defined 5 large gene expression subclasses using the Illumina BeadChip technology based on 5641 signature genes. The transcriptional profile of the 5 subclasses was driven by the underlying and most commonly described driver mutations [*NF2/SMARCB1, KLF4/TRAF7, PI3K/TRAF7*, Hedgehog (*SUFU/PRKAR1A*), and *POLR2A*]. Fourteen out of our 18-GEP were present in their gene list with most of the genes (8/14) belonging to the *NF2/SMARCB1* [44]. Finally, *LAMP5* (encoding a cellular membrane component) and, *FMO2* (encoding an NADPH-dependent enzyme; a flavin-containing monooxygenase family member) have not been previously described in meningioma to the best of our knowledge.

While our study is limited by the lack of confirmatory gene expression level by another method and by protein expression data, prior reports of the genes in our signature generally support our findings at the mRNA level. These data support the concept that angiogenesis and cell cycle regulation are important pathways in meningioma growth and progression. Another potential limitation of the study is the fact that the validation cohort contained more WHO grade II tumors than generally described (52% of samples were WHO grade tumors) and no WHO grade III tumors. Although this was not intentional and was the result of technical issues (tissue availability, nucleic acid quality, etc) this represents potential selection bias that might interfere with the results and conclusions drawn; however, training was performed on a larger, more robust and proportional dataset comprising all WHO grades. Ultimately, our study does combine one of the largest datasets of primary, non-treated meningioma tumors (n=189) with comprehensive machine learning predictive modeling. Since the data contains samples from different patient populations (i.e. different institutions) we believe this model is likely generalizable. This expression predictor could potentially be applied to patients at initial diagnosis in order to predict recurrence and more accurately guide the decision for follow-up interval or adjuvant RT. In an era of molecular profiling we should not hesitate to apply these models in the clinic in order to offer a maximum of prognostic information for clinical management. A combination of current prognostic factors (i.e. patient’s symptomatology and tumor size, tumor morphology, WHO grade, Simpson grade of surgical resection) with genomic-based mathematical predictive models will offer additional information with the ultimate goal to improve patient outcomes.

## MATERIALS AND METHODS

### Data collection and tissue samples

Three publicly available Affymetrix gene expression datasets (GSE9438, GSE16581, GSE43290) combining 127 primary, non-treated meningioma samples served as the training set [37, 38, 45]. The samples were carefully selected to exclude recurrent tumors, samples with incomplete clinical or follow-up data, and normal meningeal samples (Supplementary Table 2). Sixty-two new cases from M.D. Anderson Cancer Center served as the validation dataset. The study protocol was approved and carried out in accordance with institutional review board guidelines. Fresh frozen tissues from primary (non-treated) meningiomas were retrospectively identified and collected from the institutional tissue bank. The samples were selected based on tissue availability and availability of clinical information. Hematoxylin and eosin (H&E) stained slides (Supplementary File) were reviewed and the diagnosis confirmed by two experienced neuropathologists (KDA and AO). Samples were examined histologically and where appropriate, non-neoplastic elements were grossly dissected to ensure high tumor purity (>90%) prior to RNA isolation. All cases were graded per current WHO 2016 criteria which are listed in detail in Supplementary Figure 1 legend [2].

Special histological subtypes of meningioma were not omitted (one secretory and one chordoid meningioma were included in the cohort). For patients with multiple meningiomas with independent surgical resections, only one of the samples was used, based on frozen tissue availability.

Recurrence was defined and RFS was calculated as previously described [13]. Briefly, *recurrence* was established by treating physicians by serial imaging review; as either tumor recurrence following gross total resection or tumor progression (further growth) following subtotal surgical resection. Simpson grade (criteria listed in Supplementary Figure 2 legend) was determined as previously described [6]. For three patients Simpson grade was either not available or not applicable (i.e. for non-dura based meningioma) [13]. Four patients had remote histories of RT to the head for other tumors (diffuse large B-cell lymphoma, optic glioma, pituitary adenoma, and leukemia) prior to meningioma diagnosis.

### Immunohistochemistry

Immunohistochemistry with anti-pHH3 (Ser 10) rabbit polyclonal antibody (Cell Signaling Technology, Danvers, MA, catalog# 9701L, dilution 1:100) and anti-Ki-67 mouse monoclonal antibody (Agilent DAKO, Santa Clara, CA, USA, clone MIB-1, dilution 1:500) was performed as previously described [13]. Mitotic index was defined and determined as previously described [13]. Briefly, pHH3-labeled mitoses counted per 1000 meningioma tumor cells represented the mitotic index. Three mitotic index categories were defined: 0-2, 3-4, and ≥5.

### Gene expression analysis

RNA was extracted using the MasterPure™ Complete DNA and RNA Purification Kit (Epicentre, Madison, WI, USA) per manufacturer’s protocol. A total of 1 μg of total RNA per sample was processed on Affymetrix U133 plus 2.0 expression array platform (Affymetrix Inc, Santa Clara, CA, USA) with Expression Analysis Inc, Durham, NC, USA. CEL files were imported, processed, and analyzed using the JMP^®^ Genomics 7.0 software (SAS Institute Inc, Cary, NC, USA). Probeset annotation and summarization was performed using custom chip definition file (CDF) downloaded from http://brainarray.mbni.med.umich.edu/Brainarray/Database/CustomCDF/genomic_curated_CDF.asp utilized the version 11 EntrezG CDF [46].

Since one of the publicly available datasets (GSE43290) was run on the HG-U133Av2 platform and the rest of the samples, including the validation dataset, were run on the HG-U133Plus2.0 platform, only probesets on HG-U133Av2 were used for analysis (total of 11911 probesets). CEL files for both training and validation datasets were imported in JMP^®^ Genomics using the robust multi-array average (RMA) background correction method, log2 transformed, normalized by median scaling, and summarized by median polish using the custom CDF. Batch effect was present in both datasets and the batch normalization function for expression data (JMP^®^ Genomics) was applied.

### Statistical analysis

In order to correct for the increased number of censored events present in both datasets (85.83% in training dataset and 80.65% in validation dataset) the deviance residuals were calculated and used further as predictor variables for model selection. In the training dataset, the probe sets (n=491) having the MAD score ≥ 0.5 were selected. These 491 probe sets were filtered by k-means clustering using a correlation radius of 0.95 and 98 probesets that failed to cluster with this stringency were further eliminated. In order to select the probesets that best separated meningioma recurrences, the remaining 393 probe sets were introduced into a support vector machine classification method with RBM [47, 48] using the deviance residual as the continuous dependent variable. The dependent variable was standardized using an euclidean length equivalent scale and a genetic algorithm (SAS GENESELECT) was used for variable selection. Multiple models were run and the final model was chosen based on the maximum Harrell C Statistic [49] of 1, minimum Akaike and Bayesian information criteria [50] with the lowest possible RMSE. Fifty-fold cross-validation with 10% of the data holdout was run on the final selected model and the RMSE was recorded. The RBM predictor probabilities generated for each sample were normalized by inverse transformation in order to reduce outliers and homogenize the data, and a cutoff was calculated by partition analysis using the deviance residual as the dependent/predictor variable. The optimal, selected model was then independently applied to the validation dataset.

Cox uni- and multivariable analyses, Log-rank and Kaplan–Meier survival graphics were generated in R 3.1.2 GUI 1.65 Mavericks build (6833) (The R Foundation for Statistical Computing http://www.R-project.org). Wilcoxon tests were performed using JMP^®^ Genomics 7.0 software (SAS Institute Inc, Cary, NC, USA). The reported median survivals were calculated via Kaplan–Meier.

### Note

This work has been presented at the Society of Neuro-Oncology Annual Meeting, San Antonio, USA, Nov, 2015.

## SUPPLEMENTARY MATERIALS FIGURES AND TABLES







## Abbreviations

CDF: custom chip definition file
GEP: gene expression profiles
H&E: hematoxylin and eosin
MAD: median absolute deviation score
RBM: radial-basis smoothing kernel
RFS: recurrence-free survival
RMSE: root mean square error
RT: radiation therapy
WHO: World Health Organization
