# Supplementary material for: A gene expression signature predicts recurrence-free survival in meningioma
Source: Oncotarget. 2018 Feb 15;9(22):16087–98. doi: 10.18632/oncotarget.24498 (PMC5882319; doi:10.18632/oncotarget.24498)
Supplement: Supplementary file 1 [file oncotarget-09-16087-s001.pdf]

A gene expression signature predicts recurrence-free survival in meningioma

SUPPLEMENTARY MATERIALS

Hematoxylin and eosin staining protocol

Formalin-fixed paraffin embedded slides: Following deparaffinization (heat and xylene) slides were rehydrated in decreasing ethanol concentrations (100%, 95%, 70%) and placed in hematoxylin (5 minutes) followed by acid alcohol, ammonia water, and eosin (1 minute) then dehydrated in increasing ethanol concentrations (95%, 100%), placed in xylene, then coverslipped while wet.

Supplementary Table 1: Gene expression levels in both training and validation datasets per risk groups. Data is shown post-normalization.

See Supplementary File 1

Supplementary Table 2: Overview of samples in the training dataset.

See Supplementary File 2

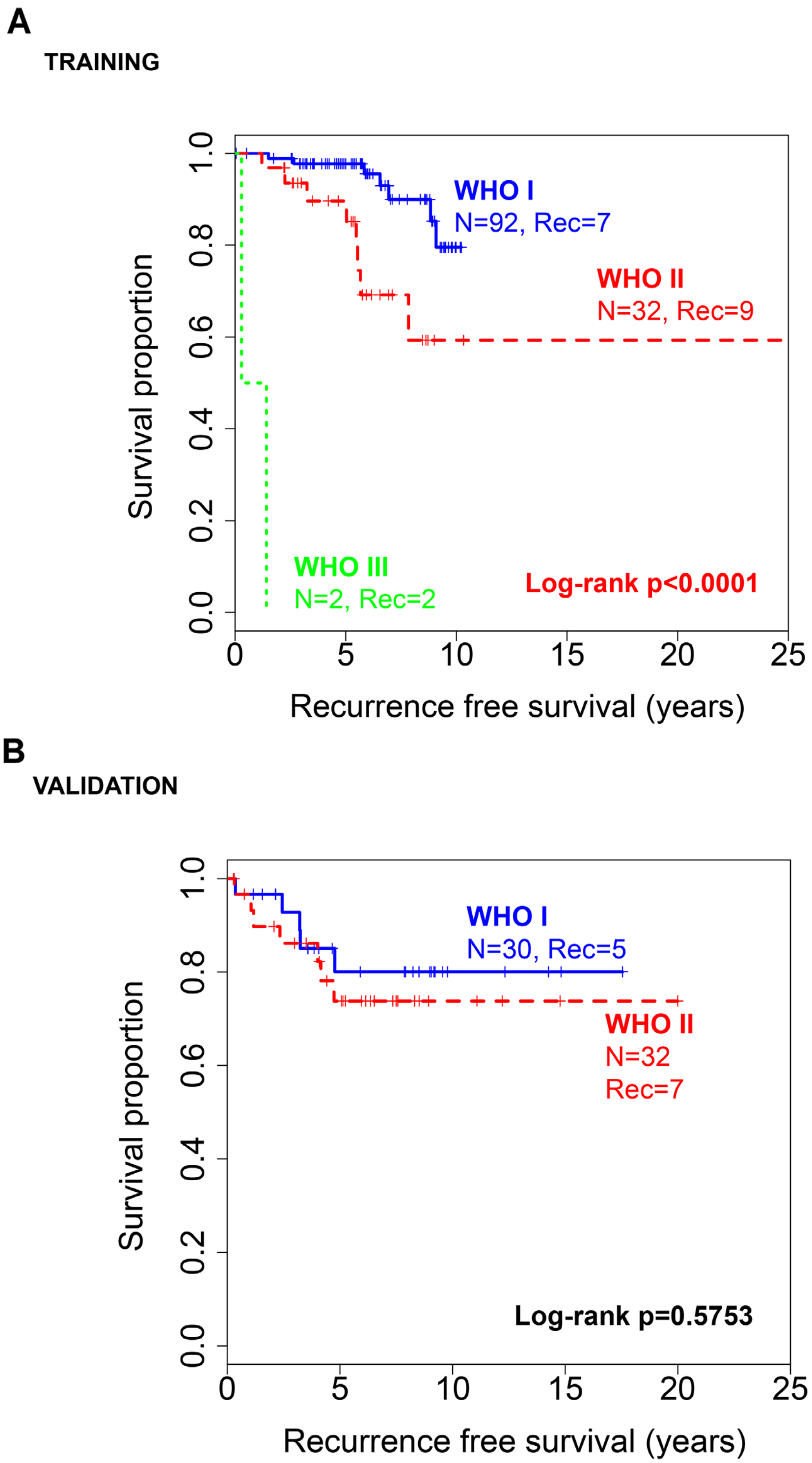

Supplementary Figure 1: Kaplan-Meier survival analysis stratified by WHO grade in the TRAINING cohort (n=126*) **(A)** and in the VALIDATION cohort (n=62) **(B)**. (Abbreviations: N – number of samples, Rec – number of recurrences, WHO – World Health Organization). *The WHO grade was missing for one case. ***Note:*** The 2016 WHO grading criteria for meningioma: **WHO grade I:** less than 4 mitoses per 10 high-power microscope fields (HPF); **WHO grade II (atypical)** (at least one of the following): >= 4 mitoses but less than 20 mitoses per 10 HPF, brain invasion, clear cell morphology, chordoid morphology, at least 3 of the following 5 morphological features (patternless growth, hypercellularity, small cell change, macronucleoli, necrosis); **WHO grade III (anaplastic)** (at least one of the following): >=20 mitoses per 10 HPF, rhabdoid morphology, papillary morphology.

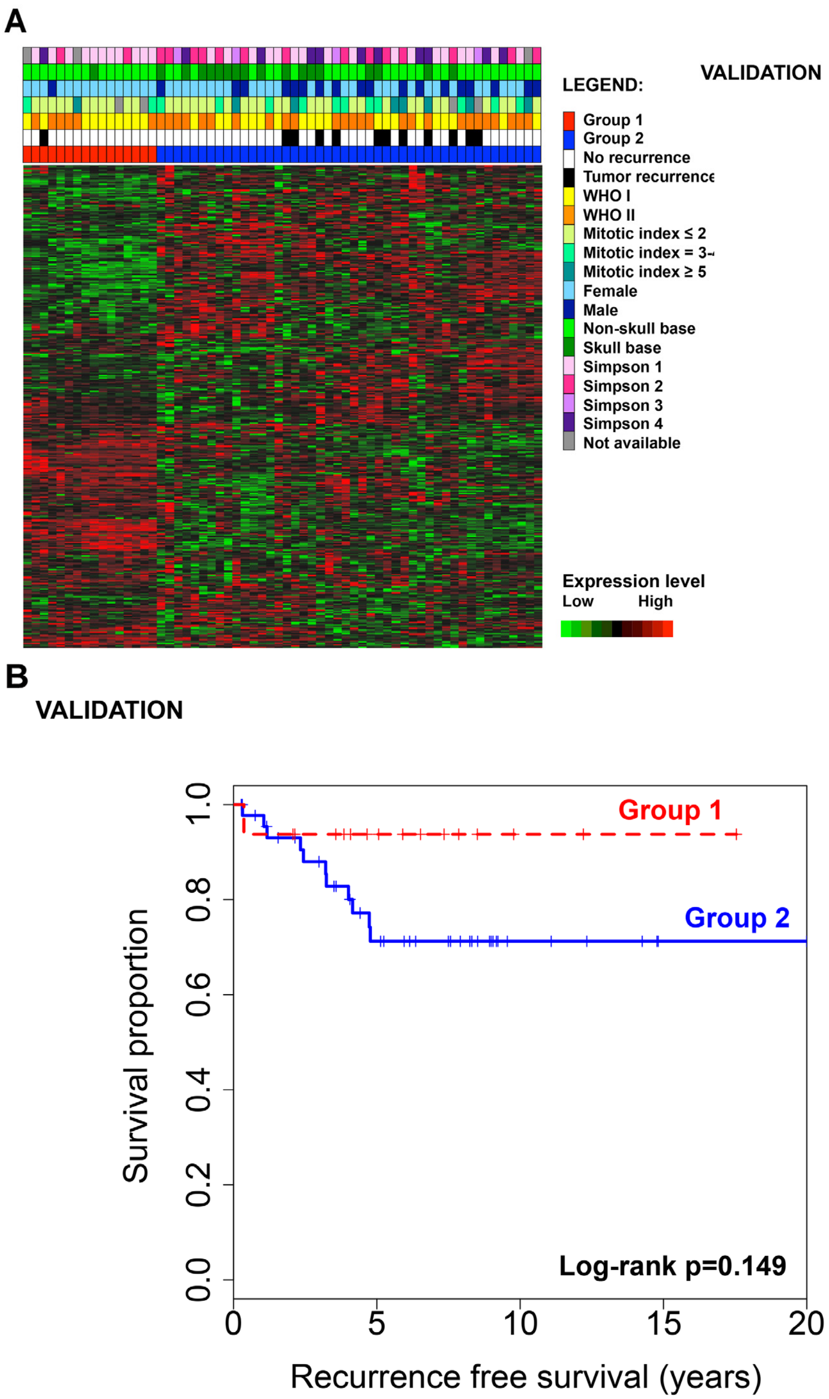

Supplementary Figure 2: Unsupervised hierarchical clustering with the initial filtered 393 probe sets in the VALIDATION dataset (n=62) separates 2 differentially expressed groups of tumors. Each row represents a probe set and each column represents a sample. Expression values are shown after batch normalization **(A)**. Kaplan-Meier survival analysis illustrates a trend for decreased tumor recurrence time for patients with meningioma from group 2 **(B)**. ***Note:*** Simpson grading criteria for meningioma surgical resection: **1** – Gross total resection of meningioma with excision of its dural attachment or involved bone/sinus; **2** - Gross total resection of meningioma with coagulation/cauterization/charring of its dural attachment; **3** - Gross total resection of meningioma without resection or cauterization of its dural attachments or involved bone/sinus; **4** – Subtotal resection of meningioma; **5** – Decompression with or without biopsy.
